# Frequency of Pulmonary Hypertension and Its Associated Risk Factors in End-Stage Renal Disease (ESRD) Patients on Maintenance Hemodialysis

**DOI:** 10.7759/cureus.55206

**Published:** 2024-02-29

**Authors:** Manisha Khemchandani, Kiran Nasir, Ruqaya Qureshi, Murtaza Dhrolia, Aasim Ahmad

**Affiliations:** 1 Nephrology, The Kidney Centre Post-Graduate Training Institute, Karachi, PAK

**Keywords:** pulmonary hypertension, chronic heart failure, pulmonary artery systolic pressure, maintenance hemodialysis, end stage renal disease (esrd)

## Abstract

Introduction

Pulmonary hypertension (PH) is a recognized complication in patients with end-stage renal disease (ESRD undergoing maintenance hemodialysis (MHD). PH is commonly found in patients with chronic kidney disease (CKD) and ESRD. PH is associated with increased morbidity and mortality in patients with CKD.

Methodology

This cross-sectional study aimed to assess the prevalence of PH and its associated risk factors in MHD patients. A total of 220 ESRD patients on MHD patients at The Kidney Center, Karachi, Pakistan, aged 18-70 were included. Patients with chronic obstructive lung disease, valvular heart disease, and obstructive sleep apnea were excluded, as these conditions can be responsible for PH. PH was evaluated by echocardiography (ECHO), which was performed by a cardiologist.

Results

The mean age was 50.65 ± 14.4 years, with 131 (59.5%) males and 89 (40.5%) females. The average duration on hemodialysis was 5.3 ± 2.8 years. Hypertension (89.5%) and ischemic heart disease (24.1%) were prominent comorbidities. Hypertensive nephropathy (42.7%) was the leading cause of ESRD. Left ventricular hypertrophy was mild in most cases (85.5%), whereas regional wall motion abnormality (RWMA) was common (67.3%). The average pulmonary artery pressure was 35.2 ± 15.3 mmHg. Out of 220 patients, 109 patients (49.8%) of them had mild PH, nine patients (4.1%) had severe PH, and 72 patients (32.7%) had moderate PH. Associations between PH and various factors were examined. RWMA, left ventricular hypertrophy, and left ventricular ejection fraction were significantly associated with PH (p < 0.001). Serum calcium and albumin levels were also associated with PH severity (p < 0.05). Other demographic and laboratory parameters did not show a significant association.

Conclusion

This study highlights the prevalence of PH in MHD patients and identifies associated risk factors. Understanding these associations can aid in better managing PH in ESRD patients.

## Introduction

Chronic kidney disease (CKD) is a global health problem. Pulmonary hypertension (PH) is an increasingly recognized consequence of cardiovascular disease, which is a cause of morbidity and mortality in CKD and end-stage renal disease (ESRD) [1].

PH is defined as mean pulmonary artery pressure (MPAP) of more than 25 mmHg at rest or 30 mmHg during activity [2]. The World Health Organization (WHO) categorizes PH into five groups: Group 1: pulmonary arterial hypertension; Group 2: PH resulting from left heart disease; Group 3: PH caused by chronic lung disease and/or hypoxia; Group 4: chronic thromboembolic PH; and Group 5: PH resulting from unclear multifactorial mechanisms [3], with CKD patients falling into group 5 (unknown multifactorial etiology of PH) [4]. Endothelial dysfunction, increased cardiac output, and myocardial dysfunction leading to increased left ventricular filling pressure are all linked to the pathogenesis of PH. Left ventricular dysfunction accounts for the vast majority of PH in patients with CKD [4]. The prevalence of PH in maintenance hemodialysis (MHD) patients has been reported between 25% and 51% [5].

Endothelial dysfunction, vascular calcification and stiffening, volume overload, arterio-venous fistula, sleep apnea, dialysis membrane exposure, and severe anemia are risk factors for PH in CKD patients [6].

The mechanisms responsible for the development of PH are chronic hypoxia caused by anemia and abnormality in respiratory gas transport, vascular calcification with thickening of the pulmonary arteries caused by alterations in calcium and phosphorus metabolism, increased vascular tone of the pulmonary arterioles caused by decreased nitric oxide synthesis, imbalance between vasodilator/vasoconstrictor mediators, and respiratory muscle dysfunction [7].

Regardless of the underlying etiology, PH is associated with poor clinical outcomes. Echocardiography (ECHO) is the preferred screening technique for PH because it allows for the noninvasive evaluation of the right ventricular systolic pressure (RVSP) based on tricuspid regurgitation (TRV) velocity [8].

This study aims to determine the frequency of PH and its associated risk factors in patients with ESRD on MHD using ECHO.

## Materials and methods

This cross-sectional study was done on MHD patients at the dialysis unit of The Kidney Center Postgraduate Training Institute, Karachi, Pakistan, from January 2023 to June 2023.

A non-probability consecutive sampling technique was utilized to enroll the patients in the study. A sample size of 220 was computed using a 95% confidence interval and 5% margin of error, derived from the previous study reporting that 54% of the patients had PH [9].

All the patients undergoing MHD for more than three months between the ages of 18-70 years were included in the study. Those with chronic obstructive lung disease, valvular and congenital heart diseases, chronic liver disease, chest wall or parenchymal lung disease, previous pulmonary embolism, collagen vascular disease, and obstructive sleep apnea were excluded.

After getting approval from the hospital's ethical review committee (TKC-ERC Ref No # 154-NEPH-12202), the patients who agreed to participate in the study were asked to sign a written informed consent. Two hundred twenty (220) MHD patients were evaluated for PH. Their baseline demographics include age, gender, weight, height, BMI, cause of ESRD, and associated comorbid conditions (hypertension, ischemic heart disease, hypothyroidism, cerebrovascular accident). Dialysis details (vascular access, arteriovenous fistula (AVF) flow rate, residual urine volume, number of dialysis sessions per week, number of years on dialysis), lab parameters (hemoglobin, transferrin saturation, ferritin, calcium, albumin, phosphorous, parathyroid hormone (PTH)), and echocardiographic findings (ejection fraction, pulmonary artery pressure (PAP), left ventricular hypertrophy (LVH), regional wall motion abnormality (RWMA), mitral regurgitation (MR)) were noted on a preformed proforma. ECHO was performed by a cardiologist on non-dialysis day according to the American Society of Echocardiography guidelines. The participants were placed in a left lateral decubitus position, with their left arm stretched above their heads [10]. In addition to TRV and right atrial (RA) pressures, the pulmonary artery systolic pressures were determined using the Bernoulli equation. The inferior vena cava's size and the collapsibility level were used to calculate the RA pressure [11].

Depending on the presence or absence of PH, patients were divided into two groups. Those patients diagnosed with PH were further sub-classified as mild (35-50 mmHg), moderate (50-70 mmHg), or severe (> 70 mmHg) [12].

Data were entered into Statistical Product and Service Solutions (SPSS, version 23; IBM SPSS Statistics for Windows, Armonk, NY) and then analyzed. The mean and standard deviation were calculated for continuous variables of age and duration of hemodialysis sessions, along with median and interquartile range, due to skewed data. Frequency and percentages were calculated for categorical data. Age, gender, and duration of hemodialysis sessions were analyzed by stratification. Association between variables was assessed by chi-square or Fisher’s exact test as appropriate. A p-value of 0.05 was considered statistically significant.

## Results

We enrolled 220 patients in our study, with a female-to-male ratio of 1:1.5. The mean age of the participants was 50.65 years (standard deviation: 14.4 years). There were 131 male patients (59.5%) and 89 female patients (40.5%). On average, the participants have been on MHD for 5.3 years (standard deviation: 2.8 years). Hypertension was the most prevalent comorbid condition in these patients (197, 89.5%), followed by ischemic heart disease (IHD, 53, 24.1%). Small size kidneys with hypertension were the most frequent cause of ESRD (94, 42.7%). LVH was mild in most of the cases (188, 85.5%); on the contrary, RWMA was common in our patients (148, 67.3%). The average PAP among the patients was 35.2 mmHg (standard deviation: 15.3 mmHg). The demographic characteristics of the study population are described in Table 1.

**Table 1:** Baseline demographic and clinical characteristics of the patients (n = 220) STD = standard deviation; n = number of patients; mL = milliliters; AVF = arteriovenous fistula

		Mean ± STD, n (%)
Age (in years)		50.65 ± 14.4
Gender	Male	131 (59.5)
	Female	89 (40.5)
Years on hemodialysis		5.3 ± 2.8
Comorbid condition	Hypertension	197 (89.5)
	Cerebrovascular accident	9 (4.1)
	Ischemic heart disease	53 (24.1)
	Hypothyroidism	14 (6.4)
Positive smoking history		72 (32.7)
Cause of end-stage renal disease	Diabetes mellitus	83 (37.7)
	Hypertension	94 (42.7)
	Glomerulonephritis	25 (11.4)
	Adult polycystic kidney disease	5 (2.3)
	Obstructive Uropathy	13 (5.9)
Vascular access	Arteriovenous fistula	200 (90.9)
	Temporary catheter	20 (9.1)
Left ventricular hypertrophy	Mild	188 (85.5)
	Moderate	31 (14.1)
	Severe	1 (0.5)
Regional wall motion abnormality		148 (67.3)
Residual urine (in mL/24 hours)		53.6 ± 142.8
AVF flow rate		329.5 ± 50.8
Left ventricular ejection fraction		47.8 ± 9.7
Pulmonary artery pressure		35.2 ± 15.3

Laboratory parameters are described in Table 2.

**Table 2:** Laboratory parameters of the patients STD = standard deviation; IQR = interquartile range

Laboratory parameters	Mean ± STD	Median, IQR
Calcium	8.4 ± 0.83	8.3, 0.94
Hemoglobin	11.2 ± 1.3	11.2, 1.8
Transferrin saturation	30.8 ± 15.3	11.2, 16
Ferritin	606 ± 695.9	413, 521.8
Phosphorus	4.7 ± 1.6	4.5, 1.9
Albumin	3.5 ± 0.43	3.6, 0.5
Parathyroid hormone	554.9 ± 538.6	371.5, 510.5

**Figure 1:**
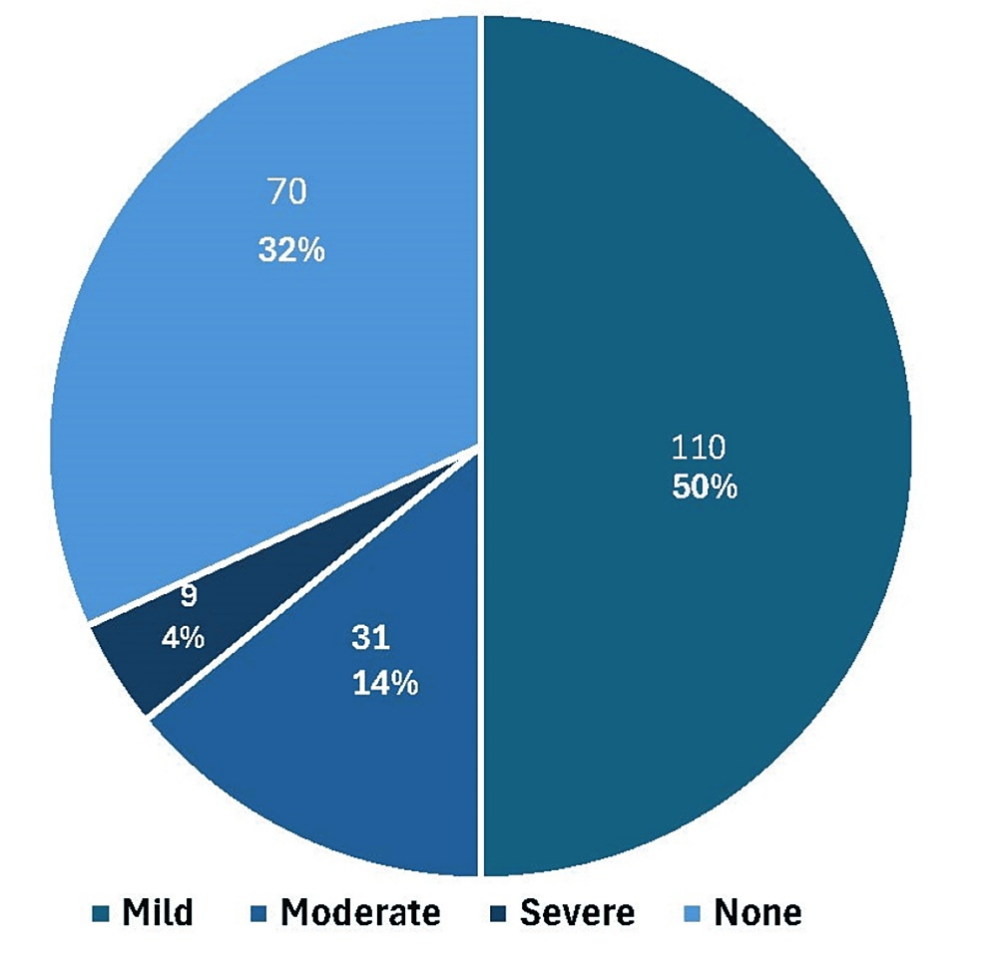
Frequency of pulmonary hypertension in the study population

Most of our patients had mild PH (110, 50%), while 9 (4%) patients suffered from severe PH (Figure 1).

The chi-square test results for the association between these variables and the severity of PH indicate that RWMA (p < 0.001), LVH (p < 0.001), and LVEF (p < 0.001) were significantly associated with the severity of PH. However, gender (p = 0.71), hypertension (p = 0.819), cerebrovascular accident (p = 0.331), ischemic heart disease (p = 0.53), hypothyroidism (p = 0.788), and smoking (p = 0.778) did not show significant association with the severity of pulmonary hypertension in this study. We estimated that the majority of the patients who had mild PH were those who also had RWMA as compared to no RWMA (74.55 v/s 25.5%, respectively). RWMA was more prevalent in those patients who had moderate and severe PH (80.6% and 88.9%, respectively). We found that the patients with no PH had maximum LVEF (51.4 ± 7.4) as compared to patients with severe PH (35.6 ± 11.8; Table 3).

**Table 3:** Association of demographic and clinical variables with pulmonary hypertension

Variables		Mild (110, 50%)	Moderate (31, 14.9%)	Severe (9, 4.1%)	None (70, 31.8%)	P-value
Gender	Male	68 (61.8)	17 (54.8)	4 (44.4)	42 (60)	0.71
	Female	42 (38.2)	14 (45.2)	5 (55.6)	28 (40)	
Hypertension	No	10 (9.1)	3 (9.7)	1 (11.1)	9 (39.1)	0.819
	Yes	100 (90.9)	28 (90.3)	8 (88.9)	61 (87.1)	
Cerebrovascular accident	No	103 (93.6)	31 (100)	9 (100)	68 (97.1)	0.331
	Yes	7 (6.4)	0	0	2 (2.9)	
Ischemic heart disease	No	81 (73.6)	22 (71)	8 (88.9)	56 (80)	0.533
	Yes	29 (26.4)	9 (29)	1 (11.1)	14 (20)	
Hypothyroidism	No	102 (92.7)	30 (96.8)	8 (88.9)	66 (94.3)	0.788
	Yes	8 (7.3)	1 (3.2)	1 (11.1)	4 (5.7)	
Smoking	No	73 (66.4)	19 (61.3)	6 (66.7)	50 (71.4)	0.778
	Yes	37 (33.6)	12 (38.7)	3 (33.3)	20 (28.6)	
Regional wall motion abnormality	No	28 (25.5)	6 (19.4)	1 (11.1)	37 (52.9)	<0.001
	Yes	82 (74.5)	25 (80.6)	8 (88.9)	33 (47.1)	
Left ventricular hypertrophy	Mild	93 (84.5)	21 (67.7)	5 (55.6)	69 (98.6)	<0.001
	Moderate	16 (14.5)	10 (32.3)	4 (44.4)	1 (1.4)	
	Severe	1 (0.9)	0	0	0	
Left ventricular ejection function		48.6, 8.7	40.3, 11.1	35.6, 11.8	51.4, 7.4	<0.001

The lack of significance suggests that the duration of years on hemodialysis does not vary significantly across the different groups in our study. Among all laboratory parameters, serum calcium and albumin were found to be significantly associated with the severity of pulmonary hypertension (p < 0.05). We detected that the patients without PH had higher calcium and albumin as compared to the patients who had any degree of PH (8.5 ± 0.7 and 3.7 ± 0.4, respectively; Table 4).

**Table 4:** Association of lab parameters with the severity of pulmonary hypertension

Lab parameters	Mild	Moderate	Severe	None	P value
Hemoglobin	11.2 ± 1.3	11.1 ± 1.3	10.4 ± 1.8	11.2 ± 1.3	0.36
Transferrin saturation	31.1 ± 14.1	31.2 ± 20.7	27.9 ± 18.9	30.3 ± 13.7	0.48
Ferritin	725.8 ± 885.4	539.1 ± 419.5	344.8 ± 231.3	483.3 ± 408.6	0.26
Calcium	8.2 ± 0.7	8.7 ± 0.9	8.2 ± 1.7	8.5 ± 0.7	0.037
Phosphorus	4.6 ± 1.6	4.4 ± 1.4	5.5 ± 1.4	4.9 ± 1.7	0.197
Albumin	3.5 ± 0.4	3.5 ± 0.4	3.5 ± 0.6	3.7 ± 0.4	0.01
Parathyroid	570.4 ± 533.2	440.1 ± 341.3	581.1 ± 714	578.1 ± 595.8	0.741

## Discussion

Our study observed the prevalence of PH and pointed out the risk factors that contribute to it in patients with ESRD on MHD using ECHO as the diagnostic tool.

The findings demonstrated a high frequency of PH among MHD patients. Out of 220 patients, 150 (68.18%) of our study population had PH, which is high in comparison to studies performed by Suresh et al. (PH 43.5%) [1], Tudoran et al. (PH 52.94%) [7], Navaneethan et al. (PH 21%) [13], and Sonkar et al. (PH 25.6%) [14].

Out of 220 participants in our study, 109 (49.8%) had a diagnosis of mild PH, demonstrating that this condition affects a substantial portion of MHD patients. Nine (4.1%) patients had severe PH, and 72 (32.7%) of patients had moderate PH. These results are in contrast to the study performed by Sonkar et al. [14] who reported mild PH in seven (33.3%) and moderate PH in 14 (66.7%) and Suresh et al. [1] who found mild PH in 22 (20.4%) and moderate PH in 23 (21%). These results highlight the importance of PH screening in ESRD patients on MHD to enable early identification and suitable therapy.

Among our study population, 197 (89.5%) had systemic hypertension. Itelman et al. evaluated the association of pulmonary hypertension with systemic hypertension among patients with apparently normal left ventricular diastolic function. In their study, compared to normotensive patients, hypertensive patients were 3.2 times more likely to have PH (95% CI: 2.91-3.53; p < 0.001) [15]. These results are similar to studies done by Suresh et al. [1], Tudoran et al. [7], and Sonkar et al. [14], which showed hypertension as a prevalent comorbidity in ESRD patients, which may have a role in the development of PH.

The severity of PH was substantially correlated with the echocardiographic findings of RWMA, LVH, and LVEF. In our study, the prevalence of RWMA was higher in patients with mild PH (74.55%), moderate PH (80.6%), and severe PH (88.9%). Furthermore, LVEF was considerably lower in patients with severe PH (35.6 ± 11.8) compared to those without PH (51.4 ± 7.4). These findings are comparable to a study conducted by Navaneethan et al. [13], in which participants with PH had lower LVEF (53.1, 10.6%) and higher prevalence of LVH (64.8%, 5%) than those without PH (LVEF: 54.6, 8.0%; LVH: 47.7%). These results imply that structural and functional defects of the heart have a major impact on the development and progression of PH in patients undergoing MHD.

The levels of serum calcium and albumin were substantially correlated with the severity of PH among all laboratory variables investigated. In comparison to individuals with any level of PH, patients without PH had higher calcium and albumin levels (calcium: 8.5 ± 0.7 mg/dL, albumin: 3.7 ± 0.4 g/dL). Li et al. demonstrated similar findings in their study, showing an inverse correlation between PH and serum calcium levels [16]. There were no statistically significant associations between PH and years on HD as compared to studies performed by Chhabra et al. [17].

Several limitations must be addressed despite the fact that this study was successful in identifying several risk factors for PH in patients with ESRD on MHD. Because of the study's cross-sectional design, the results showed associations and not causative factors. The study was done in a single center of an urban city in Pakistan. More studies from different cities and centers are needed to confirm the results. Despite these limitations, our study brought attention to an important phenomenon that can have health risks for this already vulnerable population.

## Conclusions

In conclusion, employing ECHO as the diagnostic tool, this study adds valuable information about the prevalence of PH and its associated factors in patients with ESRD on MHD in our population. RWMA, LVH, and LVEF were significantly associated with the severity of PH in our study. In patients undergoing MHD, early diagnosis and intervention may be needed to improve outcomes.
